# Thoracic Epidural Anesthesia After a Transversus Abdominis Plane Block With Liposomal Bupivacaine in a Patient With Chronic Opioid Use: A Case Report

**DOI:** 10.7759/cureus.48234

**Published:** 2023-11-03

**Authors:** Rafael Arsky Lombardi, Kyle Ringenberg, Sara Amaral, Heitor Medeiros, Nicholas Heiser

**Affiliations:** 1 Anesthesiology, University of Nebraska Medical Center, Omaha, USA; 2 Anesthesiology, Hospital Regional Deputado Afonso Guizzo, Ararangua, BRA; 3 Anesthesiology, Hospital Universitário Onofre Lopes, Natal, BRA

**Keywords:** acute pain, epidural anesthesia, regional anesthesia, local anesthetic, liposomal bupivacaine

## Abstract

Liposomal bupivacaine is a long-acting local anesthetic drug that provides extended analgesia. A 45-year-old man with metastatic colon cancer and an intrathecal morphine pump for chronic pain underwent a transverse colectomy for a malignant transverse colon obstruction in this case report. The patient reported severe pain despite preoperative fascial plane blocks with liposomal bupivacaine and postoperative pain management strategies. As a result, an exploratory laparotomy was performed to rule out any underlying causes, but no new injuries were discovered. On postoperative day 1, a thoracic epidural catheter was inserted to provide better pain relief for the patient. The patient's pain was well-controlled by postoperative day 4, allowing the epidural catheter to be removed. On postoperative day 5, the patient was discharged home without complications. This case highlights the difficulties in managing post-laparotomy pain as well as the potential benefits of combining multiple analgesic modalities. It also emphasizes the pharmacokinetic properties of liposomal bupivacaine, emphasizing the need for caution due to its prolonged systemic presence and potential for systemic anesthetic toxicity.

## Introduction

Liposomal bupivacaine is a multivesicular formulation designed for the prolonged release of bupivacaine, providing analgesia for up to 72 hours following a single dose [1,2]. It is approved by the United States Food & Drug Administration (FDA) for surgical infiltration, fascial plane blocks, and interscalene brachial plexus blocks. The administration of liposomal bupivacaine results in systemic plasma levels of bupivacaine, which can persist for 96 hours after local infiltration. The manufacturer recommends avoiding additional use of local anesthetics within that timeframe [2,3].

The patient provided written Health Insurance Portability and Accountability Act (HIPAA) authorization to publish this case report. This article adheres to the CARE (CAse REport) guidelines for case reports [4].

This article was previously presented as a meeting abstract at the 2023 ASRA Spring Meeting on April 23, 2023.

## Case presentation

A 45-year-old male with metastatic colon cancer and cancer-related chronic pain treated with an intrathecal morphine pump presented with acute abdominal pain caused by a malignant transverse colon obstruction with cecum dilation. The patient's intrathecal pump was programmed with a morphine infusion rate of 2 mg/day, supplemented by a personal therapy manager (PTM - intrathecal demand dose), delivering 0.7 mg over 10 minutes, with a maximum frequency of six times per day.

Admission for exploratory laparotomy with a midline incision resulted in a transverse colectomy and a right colostomy. The patient had a rapid-sequence induction with fentanyl 250 mcg, ketamine 120 mg, and succinylcholine 120 mg. After intubation, he received rocuronium 70 mg and was maintained with sevoflurane 2%, FiO_2 _(fraction of inspired oxygen) 50%, and lactate Ringer infusion of 2500 ml. Hypotension was managed with phenylephrine boluses. Hydromorphone 2 mg was administered 30 minutes before extubation. Bilateral rectus sheath (RS) and lateral transversus abdominis plane (TAP) blocks were placed on pre-emergence using 20 cc of liposomal bupivacaine 1.3% (266 mg) diluted in 30 cc of preservative-free bupivacaine 0.25%. A total of 50 cc was divided evenly among injection sites.

After extubation, the patient was transported to the post-anesthesia care unit, where he complained of excruciating abdominal pain with a numerical rating scale (NRS) of 10/10. Additional intravenous doses of fentanyl (100 μg), hydromorphone (1 mg), and ketamine (10 mg) provided only modest relief.

On POD 0, we initiated intravenous PCA with 1 mg of hydromorphone every 10 minutes with a baseline infusion of 2 mg/h and hydromorphone 2 mg/h as needed for breakthrough pain. This regimen marginally enhanced the patient's pain scores. In addition, we prescribed a continuous infusion of 0.15 mg kg^-1^ h^-1 ^ketamine, which provided additional but insufficient relief. Consequently, the PCA dosage was modified to 1.5 mg every 10 minutes, and the basal rate was increased to 3 mg/h.

Nevertheless, the patient endured excruciating pain, leading to a subsequent exploratory laparotomy. During this procedure, no new findings were discovered. After discussing with the surgical and nursing teams, the multidisciplinary team determined that he should remain intubated overnight while the hydromorphone and ketamine infusions remained unchanged.

After extensive discussions with the surgical team and the patient's family regarding the risks, benefits, and various analgesic options, paying distinctive consideration to the potential risk of local anesthetic systemic toxicity (LAST), we inserted a thoracic epidural catheter for enhanced pain management the next day (POD 1). The procedure was accomplished in the intensive care unit. The catheter was inserted into the T7-T8 space in the left lateral decubitus until loss of resistance at 7 cm. It was secured at 14 cm instead of 12 cm to ensure a safety margin for potential displacement or accidental removal. After a negative test dose of lidocaine 1.5% with epinephrine, patient-controlled epidural analgesia (PCEA) was instituted using bupivacaine 0.1% at a basal rate of 8 ml/h and a bolus dose of 2 ml every 10 minutes. Since it had been less than 96 hours since liposomal bupivacaine administration, we decided not to administer a larger bolus dose concerning potential LAST. He was extubated shortly after the procedure, and a reassessment two hours later revealed moderate pain and anesthesia levels between dermatomes T8 and T10. An additional epidural bolus of 0.5% lidocaine was administered, disseminating anesthesia to the T8-L1 levels. The ketamine infusion was consequently reduced to 0.1 mg kg-1 h. The Chronic Pain Service increased the intrathecal morphine infusion from 2 to 2.2 mg daily.

On POD 2, the patient reported a remarkable improvement in pain levels, with a numerical rating scale (NRS) of 4/10 and anesthesia levels now between T8 and L1. The basal epidural rate was adjusted to 10 ml/h to enhance pain management, effectively expanding anesthesia spread to T7-L1 and improving pain control with an NRS of 3/10. Accordingly, both infusion and demand doses for hydromorphone PCA were decreased, along with the ketamine infusion. On the same day, his oral intake was advanced to a clear liquid diet, allowing the assistant practitioners (APs) to initiate oral acetaminophen 1 g every six hours and gabapentin 100 mg every eight hours.

On POD 3, he reported mild pain, with epidural anesthesia levels spanning T6-L1. There was a significant reduction in the requirements for PCEA and intravenous hydromorphone PCA boluses. The ketamine infusion was further reduced from 0.1 to 0.08 mg · kg^-1^ · h^-1^.

On POD 4, the patient's pain was adequately managed, ultimately leading to the removal of the epidural catheter and the discontinuation of the PCEA and hydromorphone PCA demands. He was administered oxycodone 20 mg every three hours as needed and hydromorphone 1 mg intravenously every two hours for breakthrough pain. He was instructed on adequately using the PTM intrathecal pump in the event of severe pain and was discharged home the following day, exhibiting no symptoms or signs of LAST during their hospitalization.

## Discussion

Laparotomies are notorious for inducing considerable pain, and insufficient pain management has been associated with increased morbidity and mortality rates [5]. Regional anesthesia techniques have demonstrated promising outcomes in reducing postoperative nausea, vomiting, respiratory complications, and acute and chronic pain. Therefore, it is crucial to ensure optimal analgesia to improve patient safety and overall comfort [6].

For abdominal surgery, TAP and RS fascial plane blocks are frequently used. Intra-abdominal surgery causes a combination of somatic and visceral pain. Through the autonomic nervous system, sympathetic fibers near the viscera transmit visceral pain. Although abdominal wall blocks decrease somatic pain, they cannot access the paravertebral sympathetic chains responsible for transmitting visceral pain. As such, they should be employed with a multimodal pain management strategy [7].

We acknowledged that thoracic epidural anesthesia is more effective at managing postoperative pain than abdominal wall blocks; however, we did not choose it as the primary method of pain management in this particular instance. This decision was influenced by the patient's deteriorating physical condition and concerns regarding potential complications in someone with an intrathecal catheter, such as hypotension and accidental dural puncture [8].

The pharmacokinetics of liposomal bupivacaine are characterized by an initial peak occurring within one hour of drug administration, primarily due to extra-liposomal bupivacaine HCl. This initial peak is followed by a second peak between 12 and 36 hours later, which is dose-dependent and the result of the extended-release bupivacaine encapsulated within multivesicular liposomes [8]. The total dose, administration route, and vascularity at the site of drug administration influence the rate of systemic absorption of bupivacaine [3].

Despite significant inter-individual variations in plasma concentrations that can lead to systemic toxicity, extensive studies with adult human volunteers clearly show that the threshold for CNS toxicity for the total (including protein-bound and unbound) bupivacaine, levobupivacaine, and ropivacaine ranges from 2000-3000 ng/ml. The threshold for the unbound forms of these drugs is 10-20 ng/ml [9,10].

In Hu et al.'s pharmacokinetics study, a single dose of liposomal bupivacaine of 266 mg resulted in a peak plasma concentration of 800 ng/ml within the first hour, followed by a second peak plasma concentration ranging between 400 and 500 ng/ml 12 hours later. Following that, the concentration decreased gradually until 72 hours after administration [2]. Wulf et al. also demonstrated a maximum plasma concentration of 700 ng/ml one hour after a 25 mg thoracic epidural bupivacaine HCl 0.25% bolus. In comparison, Emanuelsson et al. found a maximum plasma concentration of 900 ng/ml in 21 healthy volunteers after a 21-hour epidural infusion of a 0.25% solution [11,12].

We administered 0.1% bupivacaine, considerably less than the concentrations in other studies. The thoracic epidural anesthesia infusion was initiated 12 hours after the second peak plasma concentration and 24 hours after the initial liposomal bupivacaine administration. Although an additive effect was possible, the probability of reaching toxic plasma bupivacaine levels was profoundly decreased.

## Conclusions

This case study highlights the insufficiencies of liposomal bupivacaine in managing postoperative pain for chronic pain patients. Insufficient relief necessitated multiple pharmacological interventions and a thoracic epidural catheter, underscoring the importance of tailored patient care and a collaborative multidisciplinary approach. Given these challenges, more research is needed on the drug's pharmacokinetics and the safety of local anesthetics following its use.
